# Male circumcision and the incidence and clearance of genital human papillomavirus (HPV) infection in men: the HPV Infection in men (HIM) cohort study

**DOI:** 10.1186/1471-2334-14-75

**Published:** 2014-02-10

**Authors:** Ginesa Albero, Xavier Castellsagué, Hui-Yi Lin, William Fulp, Luisa L Villa, Eduardo Lazcano-Ponce, Mary Papenfuss, Martha Abrahamsen, Jorge Salmerón, Manuel Quiterio, Alan G Nyitray, Beibei Lu, F Xavier Bosch, Anna R Giuliano

**Affiliations:** 1 Unit of Infections and Cancer (UNIC), Cancer Epidemiology Research Program (CERP), Catalan Institute of Oncology (ICO), IDIBELL. L'Hospitalet de Llobregat 08908, Barcelona, Spain; 2 CIBER en Epidemiología y Salud Pública, CIBERESP (Epidemiology and Public Health Biomedical Research Consortium), Madrid, Spain; 3 Program in Public Health and the Methodology of Biomedical Research, Universitat Autonoma de Barcelona (UAB). Campus Universitat Autonoma, s/n. Cerdanyola del Valles 08193, Barcelona, Spain; 4 H. Lee Moffitt Cancer Center and Research Institute, Tampa, FL 33612, USA; 5 Department of Radiology and Basic Oncology, School of Medicine, University of Sâo Paulo and School of Medical Sciences, Santa Casa de Sâo Paulo 01223-001, Brazil; 6 Instituto Nacional de Salud Publica, Cuernavaca 62100, Mexico; 7 Instituto Mexicano del Seguro Social, Cuernavaca 62140, Mexico; 8 The University of Texas, School of Public Health, Houston, TX 77030, USA

**Keywords:** Male circumcision, Genital, HPV, Incidence, Clearance

## Abstract

**Background:**

Reported associations of male circumcision (MC) with human papillomavirus (HPV) infection in men have been inconsistent.

**Methods:**

4,033 healthy men were examined every six months for a median of 17.5 months. In each study visit, exfoliated cell specimens from the coronal sulcus/glans penis, penile shaft, and scrotum were collected and combined into one sample per person for HPV DNA detection. Samples were tested for 37 HPV types. Cox proportional hazards models were used to evaluate the association between MC and the incidence and clearance of HPV infections and specific genotypes.

**Results:**

The overall incidence of new HPV infections did not differ by MC status (for any HPV, adjusted hazard ratio (aHR) 1.08, 95% confidence interval (CI) 0.91-1.27). However, incidence was significantly lower among circumcised versus uncircumcised men for HPV types 58 (p = 0.01), 68 (p < 0.001), 42 (p = 0.01), 61 (p < 0.001), 71 (p < 0.001), 81 (p = 0.04), and IS39 (p = 0.01), and higher for HPV types 39 (p = 0.01) and 51 (p = 0.02). Despite the lack of an overall association in the risk of HPV clearance by MC (for any HPV, aHR 0.95, 95% CI 0.88-1.02), median times to clearance were significantly shorter among circumcised than uncircumcised men for HPV types 33 (p = 0.02) and 64 (p = 0.04), and longer for HPV types 6 (p < 0.001), 16 (p < 0.001), and 51 (p = 0.02).

**Conclusions:**

MC is not associated with the incidence and clearance of genital HPV detection, except for certain HPV types. The use of a single combined sample from the penis and scrotum for HPV DNA detection likely limited our ability to identify a true effect of MC at the distal penis.

## Background

The majority of sexually active men and women will acquire genital human papillomavirus (HPV) infection at some point during their lifetime. In 2008, an estimated 610,000 cancers in men and women were attributed to HPV infection worldwide [1]. The natural history of cervical HPV infection is well characterized; however, little is known about genital HPV infection in men. A recent randomized controlled trial (RCT) of male circumcision (MC) in Uganda (Africa) showed that MC reduced the incidence of high-risk HPV (HR-HPV) infections and increased clearance of HR-HPV infections at the coronal sulcus [2]. However, findings regarding the role of MC in the incidence and clearance of genital HPV infections have not been consistent across studies [3-5].

Our previously published data regarding the prevalence of genital HPV among all men enrolled in the HPV Infection in Men (HIM) Study showed no overall association between MC and genital HPV infections, except for certain HPV types [6]. The purpose of this study was to determine whether MC affects the incidence and clearance of genital HPV infections in a large, multinational cohort study of healthy men in Brazil, Mexico, and the United States (USA).

## Methods

### Study population

From June 2005 through September 2009, healthy men were enrolled in an ongoing longitudinal study of HPV infection in men, the HIM cohort study. Men were recruited from the general population, universities, and organized health-care systems. Details of the study have been previously described [7]. In brief, men were included if they 1) were aged 18–70 years, 2) were residents of southern Florida, USA, Cuernavaca, Mexico, or São Paulo, Brazil, 3) had no prior diagnosis of penile or anal cancer, 4) had no prior diagnosis of genital or anal warts, 5) had no current diagnosis or symptoms of sexually transmitted infections (STIs), 6) had not participated in an HPV vaccine study, 7) reported no previous diagnosis of HIV, 8) had not been imprisoned, homeless, or received drug treatment during the past six months, 9) were willing to commit to 10 scheduled visits every six months, and 10) had no plans to relocate within the next four years. Men who were eligible to participate reviewed and signed a written informed consent form. Before study initiation, the Human Subjects Committees of the University of South Florida, the Centro de Referencia e Tratamento de Doencas Sexualmente Transmissiveis e AIDS, Brazil, and The National Institute of Public Health of Mexico approved the research protocol.

Consenting participants completed a pre-enrollment visit, an enrollment visit scheduled approximately two weeks later, and eight additional visits after enrollment that occurred every six months over a period of four years. At each study visit, participants completed an 88-item computer-assisted self-interview (CASI) which collected information regarding sociodemographic characteristics, tobacco consumption, and sexual behavior. The primary languages spoken by participants from Brazil, Mexico, and the United States were Portuguese, Spanish, and English, respectively, and the CASI was available in these languages. After the interview, men underwent a clinical examination at which time circumcision status was assessed. Participants with full or partial circumcision were considered circumcised.

### Penile and scrotal sampling

Sampling techniques have been described in detail previously [8]. Briefly, exfoliated epithelial cells from the coronal sulcus/glans penis, penile shaft, and scrotum were collected using three different saline pre-wetted Dacron swabs and combined into one sample before DNA extraction. Among uncircumcised men, the foreskin was sampled at the time of collecting the coronal sulcus/glans penis specimen. All HPV samples were stored at -80°C prior to polymerase chain reaction (PCR) analysis and genotyping. These sampling procedures were standardized across the three countries, with one clinical site per country. Staff at each site collecting specimens and conducting physical exams were either medical doctors or nurse practitioners.

### HPV analyses

The detailed protocol for HPV analysis has been previously described [8]. Briefly, DNA was extracted using the Media Kit (QIAGen, Valencia, CA, USA), according to the manufacturer’s instructions. HPV testing was undertaken by use of PCR for amplification of a fragment of the HPV L1 gene. Specimens were tested for the presence of HPV with the PGMY09/11 L1 consensus primer system [9]. HPV genotyping was conducted on all specimens, regardless of the HPV PCR result, using the Linear Array method (Roche Molecular Diagnostics, Alameda, CA, USA) [10] to detect 37 HPV types. The 13 HPV types classified as oncogenic included: 16, 18, 31, 33, 35, 39, 45, 51, 52, 56, 58, 59 and 68. Non-oncogenic HPV types: 6, 11, 26, 40, 42, 53, 54, 55, 61, 62, 64, 66, 67, 69, 70, 71, 72, 73, 81, 82, 83, 84, IS39, and 89 (CP6108) [11]. Samples were considered valid if they were positive for β-globin and/or any HPV DNA genotype.

### Statistical analysis

Differences in the distribution of sociodemographic characteristics, tobacco consumption, and sexual behavior characteristics by circumcision status were compared using the Pearson’s chi-square test. Differences in the median time to follow-up by MC status were compared using a nonparametric equality of medians test.

The classification of “any HPV” was defined as a positive test result for at least one of the 37 HPV genotypes included in the Linear Array test. HPV detection of single or multiple oncogenic HPV types was classified as “oncogenic HPV”. Similarly, HPV detection of single or multiple non-oncogenic HPV types was classified as “non-oncogenic HPV”.

HPV incidence by circumcision status was estimated according to different classifications of HPV types: any HPV, oncogenic HPV, non-oncogenic HPV, and for each specific HPV type. For each of the above analyses, only participants free of the relevant HPV type at enrollment were included. Men with concomitant incident HPV infections could contribute to several HPV classifications. Time to newly acquired HPV was estimated using the time from the date of study entry to the date of the first detection of HPV DNA, assuming a new infection arose at the date of detection. The exact 95% confidence intervals (CIs) for incidence estimates were based on the number of events modeled as a Poisson variable for the total person-months. The unit of observation was the individual participant, and each man with an incident HPV infection was counted only once during follow-up using only data from the first occurrence. Cumulative incidence of any HPV, HPV 16, oncogenic HPV, and non-oncogenic HPV types was estimated for circumcised and uncircumcised men using the Kaplan-Meier method.

Cox proportional hazards models were used to assess the association between HPV incidence and circumcision status [12]. Hazards ratios (HRs) and 95% CIs were used as measures of association for comparison of circumcised versus uncircumcised men. The proportional hazards assumption for the Cox models was tested [13], and no violations were found, except in the non-oncogenic HPV model. The non-oncogenic HPV incidence model was stratified according to age to reduce violations of the proportional hazards assumption.

HPV clearance was defined as a participant testing HPV negative at two subsequent consecutive visits following a positive HPV test result, excluding those testing HPV positive for the first time at a participant’s final visit. Time to HPV clearance was estimated by using the time at which the participant first tested positive to the date of the first negative test. Median time to clearance was estimated among incident infections using the Kaplan-Meier method for any HPV, oncogenic HPV, non-oncogenic HPV, and for each specific HPV type, according to circumcision status. Analyses were performed for each individual HPV type. Men whose HPV infections did not clear were censored in the analysis.

Cox proportional hazards models with the robust covariance matrix estimator to account for within-subject correlation were used to assess the association between HPV clearance and circumcision status [12]. Men with HPV infections, regardless of baseline HPV status, were included in the models. HRs and 95% CIs were used as measures of association for the comparison of circumcised versus uncircumcised men. The proportional hazards assumption for the Cox models was tested [13], and HPV clearance models were stratified according to age and country to reduce violations of the proportional hazards assumption.

An HPV infection was considered persistent if a man was HPV DNA-positive at two or more consecutive visits with the same specific HPV type, and an HPV infection was considered transient if a man was positive only once. When a participant missed a study visit, the results from the next visit were used.

The same variable selection procedure was used to evaluate factors associated with incidence and clearance of HPV infections. Factors that had a p-value < 0.10 were considered covariates. Backwards selection methods, with a significance threshold of 0.05, were used to identify covariates for inclusion in the final multivariable model. Candidate variables included education, marital status, smoking status, lifetime number of female sexual partners, lifetime number of male anal sex partners, number of female sexual partners in the past 3–6 months, number of male anal sex partners in the past 3 months, and six-month visit compliance status (i.e. whether the elapsed time between follow-up visits was longer than 6.5 months). Country (USA, Brazil, and Mexico) and age (categorical) were included in all models as study design factors. In addition, HPV status at baseline was a candidate variable in the HPV clearance models.

Statistical analyses were conducted using R 2.13.0 (R Development Core Team) and SAS software, version 9.2 (SAS Institute Inc., Cary, NC). Statistical tests were two-sided, with a significance threshold of 0.05.

## Results

Of the 4,074 initial HIM Study participants, 4,033 contributed valid samples for HPV DNA detection and were included in the analyses. Thus, 19 circumcised and 22 uncircumcised men (chi-square p-value = 0.03) contributed inadequate samples, as determined by both lack of β-globin detection and absence of any HPV genotype. The median duration of follow-up was 17.5 months (interquartile range [IQR], 6.9 – 31.0 months) for this analysis. There were no significant differences in the median duration of follow-up for circumcised (17.9 months) versus uncircumcised men (17.1 months) (p = 0.5 for nonparametric equality of medians test). More than 60% of men contributed at least 4 visits (IQR, 3 – 6 visits). The majority of men were uncircumcised (63.6%) (Table 1). Sociodemographic and behavioral characteristics of men varied by circumcision status. Circumcised men were generally younger (mean age: 31 vs. 33 years) and were residents of the USA (72.7%). Statistically significant differences by circumcision status were observed for marital status, sexual orientation, lifetime number of female sexual partners, number of female sexual partners in the past 3–6 months, lifetime number of male sexual partners, number of male anal sexual partners in past 3 months, self-reported diagnosis of STIs, and six-month visit compliance status (Table 1).

**Table 1:** Baseline characteristics of participants in the study cohort

	**Uncircumcised N = 2564**		**Circumcised N = 1469**		
	**N**	**% column**	**N**	**% column**	**P-value**^**a**^
**Age (years)**					**<0.0001**
Mean (SD)	33.3 (10.3)		31.0 (12.2)		
18-30	1125	43.9	841	57.2	
31-44	1125	43.9	421	28.7	
45-70	314	12.2	207	14.1	
**Country of residence**					**<0.0001**
USA	247	9.6	1068	72.7	
Brazil	1200	46.8	198	13.5	
Mexico	1117	43.6	203	13.8	
**Marital status**					**<0.0001**
Single	955	37.4	861	58.7	
Married	1040	40.7	332	22.6	
Cohabiting	374	14.6	108	7.4	
Divorced/Separated/Widowed	187	7.3	165	11.3	
**Current smoker**					0.054
No	1931	75.5	1147	78.2	
Yes	628	24.5	320	21.8	
**Sexual orientation**					**<0.0001**
MSW	2107	82.5	1285	87.8	
MSWM	162	6.3	55	3.8	
MSM	134	5.2	42	2.9	
No sex	151	5.9	82	5.6	
**Lifetime number of female sexual partners**					**0.025**
Median (SD)	6 (2047.3)		7 (78.2)		
0	253	10.6	137	9.7	
1	193	8.1	130	9.2	
2-9	1044	43.6	566	39.9	
10-19	423	17.7	246	17.3	
20-49	355	14.8	238	16.8	
50+	124	5.2	101	7.1	
**Number of female sexual partners in past 3–6 months**					**<0.0001**
Mean (SD)	1.4 (2.3)		1.3 (1.7)		
0	722	30.7	347	24.8	
1	948	40.3	690	49.3	
2	351	14.9	165	11.8	
3+	329	14.0	197	14.1	
**Lifetime number of male sexual partners**					**<0.0001**
Median (SD)	0 (71.1)		0 (25.9)		
0	2132	84.1	1334	90.9	
1	108	4.3	41	2.8	
2-9	173	6.8	55	3.7	
10+	123	4.9	37	2.5	
**Number of male anal sexual partners in past 3 months**					**0.003**
Median (SD)	0 (3.3)		0 (1.0)		
None	2360	92.9	1406	95.7	
1	75	3.0	25	1.7	
2	31	1.2	16	1.1	
3+	73	2.9	22	1.5	
**Diagnosis of STIs, ever**					**<0.0001**
No	2006	81.2	1246	86.8	
Yes	463	18.8	190	13.2	
**HPV status at baseline**					0.793
Negative	847	33.0	492	33.5	
Positive	1717	67.0	977	66.5	
**Six-month visit compliance status**					**<0.0001**
No	1584	61.8	846	57.6	
Yes	980	38.2	623	42.4	

^a^Pearson’s chi-square test was used for statistical comparison between circumcised and uncircumcised men. Some categories do not sum to the total because of missing values. Subjects with unknown values for each characteristic were not included for calculations of p-values.

NOTE: SD, standard deviation; MSW, men who have sex with women; MSWM, men who have sex with women and men; MSM, men who have sex with men, STIs, sexually transmitted infections.

### HPV incidence by MC status

**Figure 1:**
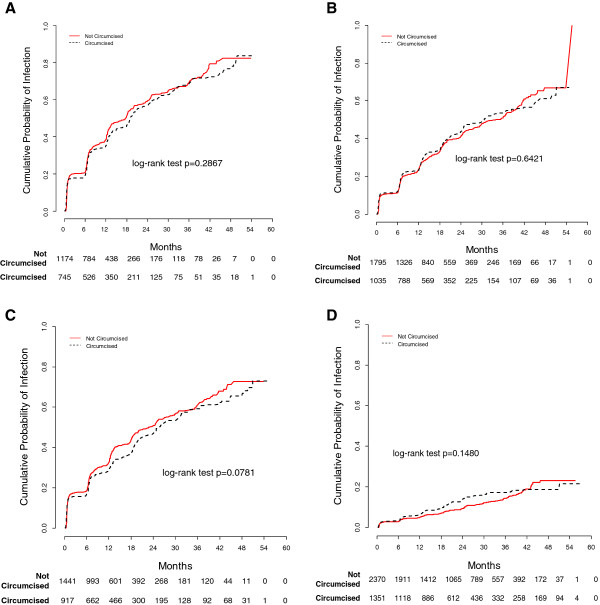
**Kaplan-Meier estimates of the cumulative incidence of human papillomavirus (HPV) infections**. Kaplan-Meier estimates of the cumulative incidence of human papillomavirus (HPV) infections for any HPV, oncogenic HPV, non-oncogenic HPV, and HPV 16 by male circumcision status: **A)** Incidence of any HPV; **B)** Incidence of oncogenic HPV; **C)** Incidence of non-oncogenic HPV; **D)** Incidence of HPV 16.

Kaplan-Meier analyses showed that the overall incidence of any HPV did not differ by circumcision status (P = 0.287, log-rank test). Similarly, incidence of HPV 16, oncogenic HPV, and non-oncogenic HPV did not differ by circumcision status (Figure 1). Estimates for incidence per 100 person-years of any HPV, oncogenic HPV, and non-oncogenic HPV were 50.5, 28.4, and 39.8, respectively, among uncircumcised men, and 45.6, 28.7, and 34.0, respectively, among circumcised men (Table 2). However, the incidence of oncogenic HPV types 39, 51, 58, and 68, and of non-oncogenic HPV types 42, 61, 71, 81 and IS39, did differ between circumcised and uncircumcised participants. Incidence was significantly higher among circumcised men than uncircumcised men for HPV types 39 (HR 1.47, 95% CI 1.11-1.94) and 51 (HR 1.30, 95% CI 1.04-1.61). In contrast, incidence was significantly lower among circumcised men than uncircumcised men for HPV types 58 (HR 0.61, 95% CI 0.43-0.88), 68 (HR 0.56, 95% CI 0.40-0.78), 42 (HR 0.59, 95% CI 0.38-0.90), 61 (HR 0.59, 95% CI 0.44-0.78), 71 (HR 0.37, 95% CI 0.21-0.65), 81 (HR 0.70, 95% CI 0.50-0.98), and IS39 (HR 0.41, 95% CI 0.20-0.86). The 12-month cumulative incidence of any HPV, oncogenic HPV, and non-oncogenic HPV among uncircumcised men was 38.7%, 23.7%, and 32.5%, respectively, compared with 35.2%, 23.3%, and 28.9%, respectively, among circumcised men.

**Table 2:** Incidence of HPV by male circumcision status

**HPV Type**	**Uncircumcised men**			**Circumcised men**			**Log-rank test p**	**HR (95% CI) [circumcised versus uncircumcised]**
	**No. Infections/No. PYs**	**Incidence per 100 PYs (95% CI)**	**% 12-month Incidence (95% CI)**	**No. Infections/No. PYs**	**Incidence per 100 PYs (95% CI)**	**% 12-month Incidence (95% CI)**		
**Any HPV**^**a**^	555/1098	50.5 (46.4, 54.9)	38.7 (35.5, 41.7)	359/788	45.6 (41, 50.5)	35.2 (31.4, 38.8)	0.29	0.93 (0.81-1.06)
**Oncogenic**^**b**^	584/2055	28.4 (26.2, 30.8)	23.7 (21.5, 25.9)	369/1285	28.7 (25.9, 31.8)	23.3 (20.5, 26)	0.64	1.03 (0.84-1.26)
**16**	204/3546	5.8 (5, 6.6)	5.1 (4.1, 6)	143/2164	6.6 (5.6, 7.8)	6.3 (4.9, 7.7)	0.15	1.17 (0.95-1.45)
**18**	97/3886	2.5 (2, 3)	1.9 (1.3, 2.5)	71/2421	2.9 (2.3, 3.7)	3.1 (2.1, 4)	0.24	1.20 (0.88-1.63)
**31**	71/3904	1.8 (1.4, 2.3)	1.6 (1.1, 2.2)	52/2462	2.1 (1.6, 2.8)	2 (1.2, 2.8)	0.40	1.17 (0.81-1.67)
**33**	24/4039	0.6 (0.4, 0.9)	0.4 (0.1, 0.6)	16/2532	0.6 (0.4, 1)	0.8 (0.3, 1.2)	0.86	1.06 (0.56-2.00)
**35**	44/3951	1.1 (0.8, 1.5)	0.8 (0.5, 1.2)	26/2492	1 (0.7, 1.5)	1 (0.5, 1.6)	0.83	0.95 (0.58-1.55)
**39**	106/3800	2.8 (2.3, 3.4)	2.6 (1.9, 3.3)	95/2358	4 (3.3, 4.9)	3.4 (2.3, 4.4)	**0.01**	**1.47 (1.11-1.94)**
**45**	94/3876	2.4 (2, 3)	1.8 (1.2, 2.4)	62/2451	2.5 (1.9, 3.2)	2.9 (1.9, 3.8)	0.70	1.06 (0.77-1.47)
**51**	187/3615	5.2 (4.5, 6)	4.5 (3.6, 5.5)	142/2160	6.6 (5.5, 7.7)	6.8 (5.4, 8.3)	**0.02**	**1.30 (1.04-1.61)**
**52**	164/3525	4.7 (4, 5.4)	5.4 (4.4, 6.4)	98/2277	4.3 (3.5, 5.2)	4.3 (3.1, 5.4)	0.80	0.97 (0.75-1.24)
**56**	79/3894	2 (1.6, 2.5)	2 (1.4, 2.7)	56/2443	2.3 (1.7, 3)	1.7 (0.9, 2.4)	0.40	1.16 (0.82-1.63)
**58**	104/3845	2.7 (2.2, 3.3)	2.5 (1.8, 3.1)	42/2467	1.7 (1.2, 2.3)	1.8 (1, 2.5)	**0.01**	**0.61 (0.43-0.88)**
**59**	165/3672	4.5 (3.8, 5.2)	3.9 (3.1, 4.8)	119/2223	5.4 (4.4, 6.4)	5 (3.7, 6.2)	0.14	1.19 (0.94-1.51)
**68**	143/3789	3.8 (3.2, 4.4)	3.9 (3, 4.7)	48/2393	2 (1.5, 2.7)	2.5 (1.6, 3.4)	**<0.001**	**0.56 (0.40-0.78)**
**Non-oncogenic**^**c**^	596/1497	39.8 (36.7, 43.1)	32.5 (29.7, 35.1)	372/1094	34 (30.6, 37.6)	28.9 (25.7, 32)	0.08	0.83 (0.69-1.00)
**6**	201/3553	5.7 (4.9, 6.5)	5.4 (4.4, 6.3)	104/2258	4.6 (3.8, 5.6)	3.9 (2.8, 5)	0.11	0.82 (0.65-1.04)
**11**	58/3961	1.5 (1.1, 1.9)	1.2 (0.7, 1.7)	25/2511	1 (0.6, 1.5)	1 (0.4, 1.5)	0.12	0.69 (0.43-1.10)
**26**	21/4050	0.5 (0.3, 0.8)	0.3 (0.1, 0.6)	6/2541	0.2 (0.1, 0.5)	0.5 (0.1, 0.8)	0.08	0.45 (0.18-1.12)
**40**	65/3941	1.6 (1.3, 2.1)	1.1 (0.6, 1.5)	44/2479	1.8 (1.3, 2.4)	1.7 (1, 2.4)	0.72	1.07 (0.73-1.58)
**42**	78/3914	2 (1.6, 2.5)	1.8 (1.2, 2.4)	29/2494	1.2 (0.8, 1.7)	0.8 (0.3, 1.3)	**0.01**	**0.59 (0.38-0.90)**
**53**	198/3630	5.5 (4.7, 6.3)	4.6 (3.7, 5.6)	110/2276	4.8 (4, 5.8)	4.4 (3.2, 5.5)	0.34	0.89 (0.71-1.13)
**54**	135/3815	3.5 (3, 4.2)	3.8 (2.9, 4.6)	90/2390	3.8 (3, 4.6)	3.6 (2.5, 4.6)	0.56	1.08 (0.83-1.42)
**55**	85/3873	2.2 (1.8, 2.7)	1.8 (1.2, 2.4)	66/2434	2.7 (2.1, 3.4)	2.5 (1.6, 3.3)	0.16	1.26 (0.91-1.74)
**61**	177/3628	4.9 (4.2, 5.7)	5 (4, 5.9)	68/2437	2.8 (2.2, 3.5)	3.2 (2.2, 4.2)	**<0.001**	**0.59 (0.44-0.78)**
**62**	199/3482	5.7 (4.9, 6.6)	5.6 (4.6, 6.6)	120/2231	5.4 (4.5, 6.4)	5.3 (4, 6.6)	0.69	0.95 (0.76-1.20)
**64**	11/4067	0.3 (0.1, 0.5)	0.2 (0, 0.4)	3/2557	0.1 (0, 0.3)	0.1 (0, 0.2)	0.18	0.43 (0.12-1.53)
**66**	150/3708	4 (3.4, 4.7)	3.8 (2.9, 4.6)	112/2261	5 (4.1, 6)	5.5 (4.2, 6.8)	0.08	1.25 (0.98-1.60)
**67**	42/4020	1 (0.8, 1.4)	0.9 (0.5, 1.3)	22/2528	0.9 (0.5, 1.3)	0.8 (0.3, 1.3)	0.59	0.87 (0.52-1.45)
**69**	9/4061	0.2 (0.1, 0.4)	0.1 (0, 0.2)	4/2556	0.2 (0, 0.4)	0.1 (0, 0.2)	0.60	0.73 (0.22-2.37)
**70**	85/3872	2.2 (1.8, 2.7)	2.3 (1.6, 2.9)	41/2467	1.7 (1.2, 2.3)	1.8 (1, 2.5)	0.20	0.78 (0.54-1.14)
**71**	64/3921	1.6 (1.3, 2.1)	1.7 (1.1, 2.3)	15/2531	0.6 (0.3, 1)	0.7 (0.2, 1.2)	**<0.001**	**0.37 (0.21-0.65)**
**72**	63/3928	1.6 (1.2, 2.1)	1.6 (1.1, 2.2)	31/2503	1.2 (0.8, 1.8)	1.3 (0.7, 2)	0.31	0.80 (0.52-1.23)
**73**	70/3923	1.8 (1.4, 2.3)	1.5 (1, 2)	41/2478	1.7 (1.2, 2.2)	1.6 (0.9, 2.3)	0.71	0.93 (0.63-1.37)
**81**	113/3774	3 (2.5, 3.6)	3.2 (2.4, 3.9)	49/2447	2 (1.5, 2.6)	2.1 (1.3, 2.8)	**0.04**	**0.70 (0.50-0.98)**
**82**	43/4022	1.1 (0.8, 1.4)	0.7 (0.4, 1.1)	34/2487	1.4 (0.9, 1.9)	1.5 (0.8, 2.1)	0.35	1.24 (0.79-1.95)
**83**	78/3864	2 (1.6, 2.5)	2 (1.4, 2.6)	45/2453	1.8 (1.3, 2.5)	1.7 (1, 2.4)	0.60	0.91 (0.63-1.31)
**84**	225/3516	6.4 (5.6, 7.3)	6 (4.9, 7)	163/2125	7.7 (6.5, 8.9)	7.6 (6.1, 9.1)	0.09	1.19 (0.97-1.46)
**89**	215/3564	6 (5.3, 6.9)	5.9 (4.8, 6.9)	140/2202	6.4 (5.3, 7.5)	5.7 (4.3, 7)	0.51	1.07 (0.87-1.33)
**IS39**	35/4004	0.9 (0.6, 1.2)	0.6 (0.3, 1)	9/2543	0.4 (0.2, 0.7)	0.2 (0, 0.5)	**0.01**	**0.41 (0.20-0.86)**

^a^Any HPV was defined as at least one of 37 HPV genotypes;

^b^Oncogenic HPV types: 16, 18, 31, 33, 35, 39, 45, 51, 52, 56, 58, 59, and 68;

^c^Non-oncogenic HPV types: 6, 11, 26, 40, 42, 53, 54, 55, 61, 62, 64, 66, 67, 69, 70, 71, 72, 73, 81, 82, 83, 84, 89 (CP6108), and IS39;

NOTE: PY, person-year; HR, hazard ratio; CI, confidence interval.

In univariate and multivariate analyses, no differences in the risk of HPV incidence by circumcision status were observed (Table 3). A total of 359 circumcised men (48.2%) had an incident infection, compared with 555 uncircumcised men (47.3%). For any HPV, no differences in incidence were found by circumcision status in univariate and multivariate analyses (adjusted hazard ratio [aHR] 1.08, 95% CI 0.91-1.27). Similarly, no differences in incidence were found by circumcision status for oncogenic HPV (aHR 1.11, 95% CI 0.94-1.31) and non-oncogenic HPV (aHR 1.11, 95% CI 0.94-1.30). The magnitude of the associations remained the same after adjustment for potential confounders, including sociodemographic characteristics and sexual behavior, or when changing the unit of analysis from men to infection (data not shown). Results were consistent when analyses were stratified by country of residence (data not shown).

**Table 3:** Univariate and multivariate hazard ratios for the association between male circumcision and incidence of genital HPV infection

	**Any HPV**				**Oncogenic HPV**				**Non-Oncogenic HPV**			
			**Univariate**^**a**^	**Multivariate**^**b**^			**Univariate**^**a**^	**Multivariate**^**c**^			**Univariate**^**a**^	**Multivariate**^**d**^
**Circumcision**	**Inf./ Total**	**%**	**HR (95% CI)**	**AHR (95% CI)**	**Inf./ Total**	**%**	**HR (95% CI)**	**AHR (95% CI)**	**Inf./ Total**	**%**	**HR (95% CI)**	**AHR (95% CI)**
**No**	555/ 1174	47.3	1.00	1.00	584/ 1795	32.5	1.00	1.00	596/ 1441	41.4	1.00	1.00
**Yes**	359/ 745	48.2	1.08 (0.92 – 1.27)	1.08 (0.91 – 1.27)	369/ 1035	35.7	1.11 (0.94 – 1.31)	1.11 (0.94 – 1.31)	372/ 917	40.6	1.09 (0.93 – 1.28)	1.11 (0.94 – 1.30)
**Total**	914/ 1919				953/ 2830				968/ 2358			

^**a**^Adjusted for age and country;

^**b**^Adjusted for country, age, marital status, lifetime number of female sexual partners, recent number of female sexual partners, recent number of male anal sex partners, and six-month visit compliance status;

^**c**^Adjusted for country, age, marital status, lifetime number of female sexual partners, recent number of female sexual partners, and recent number of male anal sex partners;

^**d**^Cox model stratified by age and adjusted for country, marital status, lifetime number of female sexual partners, recent number of female sexual partners, lifetime number of male anal sex partners, and six-month visit compliance status;

NOTE: Unit of analysis: men; Inf., infections; HR, hazard ratio; CI, confidence interval; AHR, adjusted hazard ratio.

### HPV clearance by MC status

**Figure 2:**
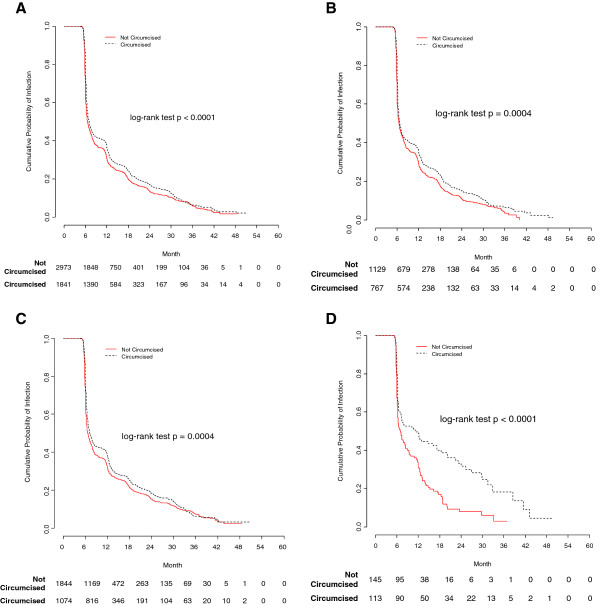
**Kaplan-Meier estimates of time to clearance of human papillomavirus (HPV) incident infections**. Kaplan-Meier estimates of time to clearance of human papillomavirus (HPV) incident infections for any HPV, oncogenic HPV, non-oncogenic HPV, and HPV 16 by male circumcision status: **A)** Clearance of any HPV; **B)** Clearance of oncogenic HPV; **C)** Clearance of non-oncogenic HPV; **D)** Clearance of HPV 16.

Kaplan-Meier analyses showed that the median time to clearance of any HPV infection was significantly longer among circumcised men than uncircumcised men (P < 0.0001, log-rank test) (Figure 2). Similarly, median time to clearance of oncogenic HPV and non-oncogenic HPV types was significantly shorter among uncircumcised men than circumcised men. Median time to clearance was also significantly shorter among uncircumcised men than circumcised men for HPV types 6 (HR 0.60, 95% CI 0.44-0.81), 16 (HR 0.56, 95% CI 0.42-0.75), and 51 (HR 0.72, 95% CI 0.54-0.95) (Table 4). Correspondingly, the probability of clearing an HPV infection was significantly lower among circumcised men compared to uncircumcised men. However, median time to clearance was significantly shorter among circumcised men compared to uncircumcised men for HPV 33 (HR 2.54, 95% CI 1.12-5.73). Although the median time to clearance for HPV 64 was significantly shorter among circumcised versus uncircumcised men, the corresponding HR did not reach statistical significance (HR 6.06, 95% CI 0.84-43.74).

**Table 4:** Clearance of HPV by male circumcision status

**HPV Type**	**Uncircumcised men**		**Circumcised men**		**Log-rank test p**	**HR (95% CI) [circumcised versus uncircumcised]**
	**New infections/Cleared infections**	**Median time to clearance (months; 95% CI)**	**New infections/Cleared infections**	**Median time to clearance (months; 95% CI)**		
**Any HPV**^**a**^	2973/2392	6.6 (6.4, 6.8)	1841/1480	7.1 (6.8, 7.3)	**<0.001**	**0.85 (0.80-0.91)**
**Oncogenic**^**b**^	1129/937	6.5 (6.3, 6.8)	767/629	6.7 (6.4, 7.1)	**<0.001**	**0.83 (0.75-0.92)**
**16**	145/118	7.1 (6.3, 8.9)	113/79	11.1 (7.2, 18.4)	**<0.001**	**0.56 (0.42-0.75)**
**18**	71/63	6.5 (6.1, 8.1)	51/44	6.3 (6.2, 7.2)	0.84	1.04 (0.71-1.54)
**31**	49/39	7.2 (6.5, 14.5)	37/31	6.4 (6.1, 6.9)	0.23	1.34 (0.83-2.17)
**33**	16/13	6.9 (5.9, 17.6)	16/16	6 (5.8, 6.6)	**0.02**	**2.54 (1.12-5.73)**
**35**	34/26	11.3 (6.5, 18.1)	18/15	10.8 (7.1, N.E.)	0.86	0.95 (0.50-1.80)
**39**	83/66	7 (6.3, 17.3)	71/56	11.2 (7.4, 12.8)	0.68	0.93 (0.65-1.32)
**45**	77/65	6.3 (6.1, 10.6)	51/48	6.1 (6.1, 6.5)	0.23	1.26 (0.87-1.84)
**51**	144/116	6.4 (6, 7.8)	117/88	8 (6.4, 13.1)	**0.02**	**0.72 (0.54-0.95)**
**52**	139/119	6.4 (6, 7.6)	84/70	6.7 (6.4, 12)	0.20	0.82 (0.61-1.11)
**56**	58/45	6 (6, 8.9)	44/41	6.3 (6, 7.9)	0.36	1.21 (0.79-1.86)
**58**	72/59	6.9 (6, 11.4)	34/26	7.1 (6.2, 12.9)	0.20	0.73 (0.46-1.17)
**59**	122/103	6.2 (6, 6.7)	89/73	6.4 (6.2, 8.5)	0.11	0.78 (0.58-1.06)
**68**	119/105	6.2 (6, 6.8)	42/42	6.4 (6.2, 10.4)	0.91	0.97 (0.68-1.39)
**Non-oncogenic**^**c**^	1844/1455	6.7 (6.4, 6.9)	1074/851	7.2 (6.9, 7.8)	**<0.001**	**0.86 (0.79-0.93)**
**6**	156/142	6.1 (6, 6.4)	80/61	7.8 (6.5, 12.5)	**<0.001**	**0.60 (0.44-0.81)**
**11**	37/27	7.8 (6.2, 14)	19/15	6.6 (6, 18.1)	0.54	1.22 (0.65-2.31)
**26**	17/14	6 (5.9, 7.1)	5/5	6.1 (6, N.E.)	0.62	0.76 (0.27-2.13)
**40**	47/41	6.4 (6, 7.8)	34/27	6.5 (6.2, 17.6)	0.09	0.66 (0.40-1.08)
**42**	63/53	9.1 (6.4, 12.3)	21/18	7.8 (6.2, N.E.)	0.96	1.02 (0.59-1.75)
**53**	150/123	6.7 (6.3, 7.8)	88/68	7.5 (6.2, 12.5)	0.10	0.78 (0.58-1.05)
**54**	111/86	8.3 (6.4, 12.1)	70/56	6.9 (6.4, 13.1)	0.93	1.02 (0.72-1.42)
**55**	67/53	7.2 (6.2, 12)	50/37	7 (6.6, 18.2)	0.20	0.76 (0.50-1.16)
**61**	126/89	6.5 (6.1, 8.5)	48/43	6.9 (6.4, 13.3)	0.86	0.97 (0.67-1.40)
**62**	148/104	7.9 (6.5, 11.8)	94/73	7.9 (6.7, 12.3)	0.88	1.02 (0.76-1.38)
**64**	8/8	6 (5.9, N.E.)	2/2	5.7 (5.5, N.E.)	**0.04**	6.06 (0.84-43.74)
**66**	110/90	6.8 (6.2, 8.5)	87/69	7.1 (6.5, 11.9)	0.49	0.89 (0.65-1.23)
**67**	35/32	6 (6, 7.2)	19/19	6.3 (6, 7.4)	0.38	1.28 (0.71-2.31)
**69**	9/9	6 (5.7, N.E.)	2/2	7.1 (6.2, N.E.)	0.99	0.98 (0.20-4.93)
**70**	62/53	6.8 (6.2, 11.7)	32/28	6.7 (6.2, 12)	0.54	1.15 (0.73-1.83)
**71**	54/39	7.5 (6.2, 13)	14/9	7.5 (6, N.E.)	0.53	0.79 (0.38-1.64)
**72**	49/43	6.1 (6, 7.4)	25/23	6.2 (6, 7.1)	0.35	1.28 (0.76-2.17)
**73**	49/39	6.2 (6, 9.2)	35/30	7.6 (6.6, 13.6)	0.45	0.83 (0.51-1.33)
**81**	94/72	7.2 (6.4, 10.3)	39/35	6.6 (6.2, 11.5)	0.29	1.25 (0.83-1.88)
**82**	30/26	6.2 (6, 11.7)	25/21	6.4 (6.2, 14.2)	0.28	0.73 (0.40-1.32)
**83**	55/45	6.9 (6, 9.5)	32/25	8 (6.4, 13.9)	0.25	0.75 (0.46-1.22)
**84**	170/126	7.2 (6.4, 11.4)	131/91	10.4 (6.7, 13.8)	0.07	0.78 (0.60-1.03)
**89**	166/116	7.2 (6.3, 11.5)	115/88	8.4 (7.7, 12.5)	0.15	0.81 (0.62-1.07)
**IS39**	31/25	6.2 (6, 13.2)	7/6	13.1 (5.9, N.E.)	0.78	0.87 (0.35-2.12)

^a^Any HPV was defined as at least one of 37 HPV genotypes;

^b^Oncogenic HPV types: 16, 18, 31, 33, 35, 39, 45, 51, 52, 56, 58, 59, and 68;

^c^Non-oncogenic HPV types: 6, 11, 26, 40, 42, 53, 54, 55, 61, 62, 64, 66, 67, 69, 70, 71, 72, 73, 81, 82, 83, 84, 89 (CP6108), and IS39;

NOTE: Analysis of clearance was limited to incident HPV detection; NE, not estimable; HR, hazard ratio; CI, confidence interval.

Univariate and multivariate analyses for clearance are presented in Table 5. HPV clearance was significantly decreased for any HPV infection among circumcised men compared to uncircumcised men in the univariate analysis (HR 0.92, 95% CI 0.86-0.99). However, after adjustment, this difference was no longer significant (aHR 0.95, 95% CI 0.88-1.02). Similarly, there were no differences in the risk of clearance for oncogenic HPV (aHR 0.90, 95% CI 0.81-1.00) or non-oncogenic HPV (aHR 0.98, 95% CI 0.89-1.07) by circumcision status. The magnitude of the associations with MC did not change substantially for transient (Table 6) versus persistent (Table 7) HPV infections. Moreover, similar results were observed when analyses were stratified by country of residence (data not shown).

**Table 5:** Univariate and multivariate hazard ratios for the association between male circumcision and clearance of genital HPV infection

	**Any HPV**				**Oncogenic HPV**				**Non-Oncogenic HPV**			
			**Univariate**^**a**^	**Multivariate**^**b**^			**Univariate**^**a**^	**Multivariate**^**c**^			**Univariate**^**a**^	**Multivariate**^**d**^
**Circumcision**	**Inf./ Total**	**%**	**HR (95% CI)**	**AHR (95% CI)**	**Inf./ Total**	**%**	**HR (95% CI)**	**AHR (95% CI)**	**Inf./ Total**	**%**	**HR (95% CI)**	**AHR (95% CI)**
**No**	4353/ 5981	72.8	1.00	1.00	1748/ 2266	77.1	1.00	1.00	2605/ 3715	70.1	1.00	1.00
**Yes**	2548/ 3398	75.0	**0.92 (0.86 – 0.99)**	0.95 (0.88 – 1.02)	1089/ 1410	77.2	**0.86 (0.78 – 0.96)**	0.90 (0.81 – 1.00)	1459/ 1988	73.4	0.97 (0.89 – 1.05)	0.98 (0.89 – 1.07)
**Total**	6901/ 9379				2837/ 3676				4064/ 5703			

^**a**^Adjusted for age and country;

^b^Cox model stratified by country and age, and adjusted for lifetime number of female sexual partners, recent number of male anal sex partners, smoking status, HPV status at baseline, and six-month visit compliance status;

^c^Cox model stratified by country and age, and adjusted for lifetime number of female sexual partners, lifetime number of male anal sex partners, HPV status at baseline, and six-month visit compliance status;

^d^Cox model stratified by country and age, and adjusted for recent number of female sexual partners, recent number of male anal sex partners, smoking status, HPV status at baseline, and six-month visit compliance status.

NOTE: Unit of analysis: infections; Abbreviations: Inf., infections; HR, hazard ratio; CI, confidence interval; AHR, adjusted hazard ratio.

**Table 6:** Univariate and multivariate hazard ratios for the association between male circumcision and clearance of transient genital HPV infection

	**Any HPV**				**Oncogenic HPV**				**Non-Oncogenic HPV**			
			**Univariate**^**a**^	**Multivariate**^**b**^			**Univariate**^**a**^	**Multivariate**^**c**^			**Univariate**^**a**^	**Multivariate**^**d**^
**Circumcision**	**Inf./ Total**	**%**	**HR (95% CI)**	**AHR (95% CI)**	**Inf./ Total**	**%**	**HR (95% CI)**	**AHR (95% CI)**	**Inf./ Total**	**%**	**HR (95% CI)**	**AHR (95% CI)**
**No**	2806/ 4434	63.3	1.00	1.00	1106/ 1624	68.1	1.00	1.00	1700/ 2810	60.5	1.00	1.00
**Yes**	1665/ 2515	66.2	0.95 (0.88 – 1.03)	0.98 (0.90 – 1.06)	745/ 1066	69.9	0.88 (0.77 – 1.00)	0.91 (0.80 – 1.04)	920/ 1449	63.5	0.99 (0.89 – 1.10)	1.01 (0.91 – 1.13)
**Total**	4471/ 6949				1851/ 2690				2620/ 4259			

^**a**^Adjusted for age and country;

^b^Cox model stratified by country and age, and adjusted for lifetime number of female sexual partners, recent number of male anal sex partners, smoking status, HPV status at baseline, and six-month visit compliance status;

^c^Cox model stratified by country and age, and adjusted for lifetime number of female sexual partners, lifetime number of male anal sex partners, HPV status at baseline, and six-month visit compliance status;

^d^Cox model stratified by country and age, and adjusted for recent number of female sexual partners, recent number of male anal sex partners, smoking status, HPV status at baseline, and six-month visit compliance status.

NOTE: Unit of analysis: infections; Excluded 120 infections that were a combination of persistent and transient infection; Abbreviations: Inf., infections; HR, hazard ratio; CI, confidence interval; AHR, adjusted hazard ratio.

**Table 7:** Univariate and multivariate hazard ratios for the association between male circumcision and clearance of persistent genital HPV infection

	**Any HPV**				**Oncogenic HPV**				**Non-Oncogenic HPV**			
			**Univariate**^**a**^	**Multivariate**^**b**^			**Univariate**^**a**^	**Multivariate**^**c**^			**Univariate**^**a**^	**Multivariate**^**d**^
**Circumcision**	**Inf./ Total**	**%**	**HR (95% CI)**	**AHR (95% CI)**	**Inf./ Total**	**%**	**HR (95% CI)**	**AHR (95**% **CI)**	**Inf./ Total**	**%**	**HR (95% CI)**	**AHR (95% CI)**
**No**	1474/ 3102	47.5	1.00	1.00	610/ 1128	54.1	1.00	1.00	864/ 1974	43.8	1.00	1.00
**Yes**	836/ 1686	49.6	0.94 (0.84 – 1.05)	0.99 (0.88 – 1.12)	324/ 645	50.2	0.76 (0.64 – 0.91)	0.84 (0.70 – 1.01)	512/ 1041	49.2	1.07 (0.92 – 1.25)	1.10 (0.94 – 1.29)
**Total**	2310/ 4788				934/ 1773				1376/ 3015			

^**a**^Adjusted for age and country;

^b^Cox model stratified by country and age, and adjusted for lifetime number of female sexual partners, recent number of male anal sex partners, smoking status, HPV status at baseline, and six-month visit compliance status;

^c^Cox model stratified by country and age, and adjusted for lifetime number of female sexual partners, lifetime number of male anal sex partners, HPV status at baseline, and six-month visit compliance status;

^d^Cox model stratified by country and age, and adjusted for recent number of female sexual partners, recent number of male anal sex partners, smoking status, HPV status at baseline, and six-month visit compliance status.

NOTE: Unit of analysis: infections; Excluded 120 infections that were a combination of persistent and transient infection; Abbreviations: Inf., infections; HR, hazard ratio; CI, confidence interval; AHR, adjusted hazard ratio.

## Discussion

MC was not associated with an overall reduction in the incidence of genital HPV detection in men. Other longitudinal studies also found no differences in the risk of HPV incidence by MC [3-5]. Lu et al. [3] and Hernandez et al. [4] used combined samples from the coronal sulcus, glans penis, shaft of the penis, and scrotum for HPV detection and found no differences in HPV acquisition by MC. Although VanBuskirk et al. [5] observed site-specific differences in HPV incidence by MC, they found no differences in overall HPV incidence by MC at any site (penile shaft, glans/corona, and scrotum). Our findings are not consistent with those from a RCT conducted in Uganda, which found that MC reduced the incidence of HR-HPV infections (rate ratio [RR] 0.67, 95% CI 0.50-0.90) [2]. However, this RCT was not comparable to our study, as they reported associations with specimens collected from only the coronal sulcus [2].

Despite the lack of evidence for an overall association, we did find higher HPV incidences rates in uncircumcised men than in circumcised men for most genotypes. These differences were statistically significant for HPV types 58, 68, 42, 61, 71, 81, and IS39. In contrast, the incidence of oncogenic HPV types 39 and 51 were significantly higher among circumcised than uncircumcised men. Consistent with our study, VanBuskirk et al. [5] reported higher HPV incidence among uncircumcised than circumcised men for >50% of the 21 specific HPV types assessed. Moreover, the RCT in Uganda found that HPV incidence was higher among uncircumcised men than among circumcised men for all HR-HPV genotypes, being statistically significant for HR-HPV types 18 and 33 [2].

No differences in overall HPV clearance by MC were observed in our study. This finding is in agreement with findings from other longitudinal studies [4,5]. VanBuskirk et al. [5] found that MC had no effect on the likelihood of detecting a persistent versus transient HPV infection over an 8-month period. Hernandez et al. [4] found that the duration of HPV infection did not vary by MC for the penile shaft, scrotum, or for all genital sites combined. However, the median duration of HPV infection at the glans/coronal sulcus was significantly longer in uncircumcised men that in circumcised men [4]. Similarly, MC increased clearance rates of HR-HPV infections at the coronal sulcus in the Uganda RCT [2]. In the study by Lu et al. [3], circumcised men were more likely to clear infection with any HPV and oncogenic HPV types using a combined HPV sample from the coronal sulcus, glans penis, shaft, and scrotum for HPV detection.

Differences in HPV clearance by MC for certain HPV types were observed in the present study. Median times to clearance for HPV types 33 and 64 were significantly shorter among circumcised men compared to uncircumcised men. The RCT in Uganda reported higher rates of clearance among circumcised men than uncircumcised men at the coronal sulcus for most HR-HPV types, but the differences were only statistically significant for HR-HPV types 39, 51, and 58 [2]. In contrast, we found that the median times to clearance for HPV types 16, 51, and 6 were significantly longer among circumcised men than uncircumcised men.

Even though our study was not designed to assess the effects of MC and HPV by anatomic genital subsite, there is evidence from the literature that the association between MC and HPV DNA detection varies according to the subsite sampled. Four out of five studies show that the effect of MC in reducing HPV infection is stronger at the glans/corona than at other, more distant, subsites [14-18]. Weaver et al. [14], found that HPV prevalence was significantly lower among circumcised men (17%) compared to uncircumcised men (32%) on the glans and foreskin. Interestingly, and consistent with our findings, when combining results from all subsites, overall HPV prevalence was similar between circumcised (31%) and uncircumcised (29%) men. Consistent with these results, a study by Nielson et al. [15] reported that HPV prevalence at the glans penis/coronal sulcus was significantly lower among circumcised men (29.8%) compared to uncircumcised men (35.2%). However, HPV prevalence combining the glans, shaft, and scrotum was similar in circumcised (48.3%) and uncircumcised men (44.8%). In another US study, Hernandez et al. [16] reported a significantly lower HPV prevalence at the glans penis/coronal sulcus among circumcised men (29%) compared to uncircumcised men (46%). However, HPV prevalence at the external genitalia was similar among circumcised (63%) versus uncircumcised (71%) men. At year 1 in the RCT in Uganda [17], HR-HPV types were more frequently detected on the coronal sulcus than on the penile shaft among both circumcised and uncircumcised men. In addition, HR-HPV prevalence at year 1 on the penile coronal sulcus was significantly lower among circumcised men (21.5%) as compared to uncircumcised men (36.3%). Finally, a study by Partridge et al. [18], conducted among male university students in the USA, found that circumcision status was not associated with incident HPV infection for any of the individual subsites or for all sites combined.

Further supporting the evidence for an effect of MC on HPV infection on the proximal region of the penis, three additional studies reported specific inverse associations between MC and HPV infection in the glans, coronal sulcus, and the distal urethra [19], the glans and coronal sulcus [20], or the urethra [21].

In addition to the subsites included for HPV DNA detection, several other factors may explain the contrasting effects observed across studies, such as differences in participants’ ages, the proportion of circumcised men, the number of HPV genotypes included in testing, the time intervals between visits, and the age at which circumcision was performed.

To our knowledge, this is the first international study reporting HPV incidence and clearance by specific HPV types in circumcised and uncircumcised men. With a sample size of 4,033 men, it is also the largest study exploring these associations. A key strength of the present study is its longitudinal design, which allowed repeated measures of genital HPV DNA status for each participant, with a median follow-up of 17.5 months and scheduled visits every six months.

Our study, despite being large, multinational, and prospective, is not free of some limitations, including: (1) the potential for misclassification of HPV infection, as we combined samples from different anatomic subsites; (2) the potential for misclassification of prevalent infections as incident infections, which may inflate the estimates of cumulative incidence; (3) the possibility of not distinguishing HPV clearance from failure to detect a true HPV infection, even though two HPV negative results following one positive result were required to define HPV clearance; (4) potentially limited generalizability, as only men willing to comply with multiple clinical visits over four years were included in the study; and (5) the potential for confounding, as there may have been relevant factors related to religious or cultural practices that may also be associated with both MC status and HPV incidence or clearance that were not taken into account.

## Conclusions

Overall, our study found that circumcision status was not associated with incidence and clearance of genital HPV detection, except for among specific HPV types. However, the use in this study of a single combined sample from the penis and scrotum likely limited our ability to identify a true effect at the distal penis. Further research is needed to determine how male circumcision impacts HPV incidence and clearance by specific anatomic subsite and HPV types.

## Abbreviations

HPV: Genital human papillomavirus; MC: Male circumcision; RCT: Randomized controlled trials; HR-HPV: High-risk human papillomavirus; HIM Study: HPV Infection in men study; STIs: Sexually transmitted infections; CASI: Computer-assisted self-interview; PCR: Polymerase chain reaction; CIs: Confidence intervals; HRs: Hazard ratios; IQR: Interquartile range; aHR: Adjusted hazard ratio; RR: Rate ratio.

## Competing interests

GA: Travel grants to conferences/meetings are occasionally granted by either GlaxoSmithKline, Merck, Sanofi Pasteur MSD, Roche or Qiagen.

GA, XC, and FXB: My research unit is involved in HPV vaccine trials organized by GlaxoSmithKline, Merck and Sanofi Pasteur MSD.

XC: Travel grants to scientific meetings and honorarium for consultancy are occasionally granted by either GlaxoSmithKline, Merck, Sanofi Pasteur MSD.

ARG: has received research grant support from Merck & Co., Inc. and GSK.

ARG and LLV: are on the speakers’ bureau for Merck and are members of its advisory board.

AGN: has received research funding from Merck & Co.

FXB: Travel grants to conferences/symposia/meetings and honorarium are occasionally granted by either GlaxoSmithKline, Merck, Sanofi Pasteur MSD, Roche or Qiagen.

All other authors declare that they have no competing interests.

## Authors’ contributions

ARG, LLV, and EL-P were the principal investigators, conceived the study, wrote the protocols, assured funding, identified clinical investigators and study personnel, and made critical revision of the manuscript. GA was responsible for conduct analysis, interpretation of data, and drafting and revision of the manuscript. WF, H-YL, and MRP made the statistical analyses, and made substantial comments to the manuscript. MA trained and supervised study clinical staff, and made substantial contributions to the manuscript. JS and MQ coordinated the field work in terms of study implementation and data collection, and made substantial comments to the manuscript. AGN, BL, XC, and FXB participated in the interpretation of data, and critical revision of the manuscript. All authors read and approved the final manuscript.

## Pre-publication history

The pre-publication history for this paper can be accessed here:

http://www.biomedcentral.com/1471-2334/14/75/prepub
